# ChemiRs: a web application for microRNAs and chemicals

**DOI:** 10.1186/s12859-016-1002-0

**Published:** 2016-04-18

**Authors:** Emily Chia-Yu Su, Yu-Sing Chen, Yun-Cheng Tien, Jeff Liu, Bing-Ching Ho, Sung-Liang Yu, Sher Singh

**Affiliations:** Graduate Institute of Biomedical Informatics, College of Medical Science and Technology, Taipei Medical University, Taipei, 110 Taiwan; Department of Life Science, College of Science, National Taiwan Normal University, Taipei, 116 Taiwan; Department of Civil Engineering, College of Engineering, National Taiwan University College of Engineering, Taipei, 106 Taiwan; Department of Clinical Laboratory Sciences and Medical Biotechnology, College of Medicine, National Taiwan University, Taipei, 100 Taiwan

**Keywords:** microRNA, Gene ontology, Chemical, Genomics, Disease

## Abstract

**Background:**

MicroRNAs (miRNAs) are about 22 nucleotides, non-coding RNAs that affect various cellular functions, and play a regulatory role in different organisms including human. Until now, more than 2500 mature miRNAs in human have been discovered and registered, but still lack of information or algorithms to reveal the relations among miRNAs, environmental chemicals and human health. Chemicals in environment affect our health and daily life, and some of them can lead to diseases by inferring biological pathways.

**Results:**

We develop a creditable online web server, ChemiRs, for predicting interactions and relations among miRNAs, chemicals and pathways. The database not only compares gene lists affected by chemicals and miRNAs, but also incorporates curated pathways to identify possible interactions.

**Conclusions:**

Here, we manually retrieved associations of miRNAs and chemicals from biomedical literature. We developed an online system, ChemiRs, which contains miRNAs, diseases, Medical Subject Heading (MeSH) terms, chemicals, genes, pathways and PubMed IDs. We connected each miRNA to miRBase, and every current gene symbol to HUGO Gene Nomenclature Committee (HGNC) for genome annotation. Human pathway information is also provided from KEGG and REACTOME databases. Information about Gene Ontology (GO) is queried from GO Online SQL Environment (GOOSE). With a user-friendly interface, the web application is easy to use. Multiple query results can be easily integrated and exported as report documents in PDF format. Association analysis of miRNAs and chemicals can help us understand the pathogenesis of chemical components. ChemiRs is freely available for public use at http://omics.biol.ntnu.edu.tw/ChemiRs.

## Background

The interactions between genetic factors and environmental factors have critical roles in determining the phenotype of an organism. In recent years, a number of studies have reported that the dysfunctions on microRNA (miRNAs), environmental factors or their interactions have strong effects on phenotypes and even may result in abnormal phenotypes and diseases [1]. Environmental chemicals have been shown to play a critical role in the etiology of many human diseases [2]. Studies have also demonstrated the link between specific miRNAs and aspects of pathogenesis [3]. The fact that a miRNA may regulate hundreds of targets and one gene might be regulated by more than one miRNAs makes the underlying mechanism of miRNA pathogenicity more complex. Many miRNA targets have been computationally predicted, but only a limited number of these were experimentally validated. Although a variety of miRNA target prediction methods are available, resulting lists of candidate target genes identified by these methods often do not overlap and thus show inconsistency. Hence, finding a functional miRNA target is still a challenging task [4]. Some integration methods and tools for comprehensive analysis of miRNA target prediction have been developed, such as miRGen [5], miRWalk [6], starBase [7], and ComiR [8]. However, it is rarely seen the consolidation and comparison of miRNA target prediction methods with chemicals, diseases, pathways and Gene Ontology (GO) related applications. Thus, it is crucial to develop the bioinformatics tools for more accurate prediction as it is equally important to validate the predicted target genes experimentally [9]. In this study, we develop a ChemiRs web server, in which various miRNA prediction methods and biological databases are integrated and relations between miRNAs, chemicals, genes, diseases and pathways are analyzed. First, we manually retrieved the associations of miRNAs and chemicals from biomedical literature, and downloaded toxicogenomics data from the comparative toxicogenomic database (CTD; http://ctd.mdibl.org) [10]. Then, our method integrated the latest versions of publicly available miRNA target prediction methods and curated databases, including DIANA-microT [11, 12], miRanda [13], miRDB [14], RNAhybrid [15], PicTar [16], PITA [17], RNA22 [18], TargetScan [19], miRWalk [6], miRecords [20], miR2Disease [21], and miRBase [22, 23]. A set of experimentally validated target genes integrated from the miRecords and mirTarBase [24] servers is also integrated in the ChemiRs server. In addition, information from KEGG [25], REACTOME [26], and Gene Ontology [27] databases were organized into ChemiRs manually. The logical restriction was also designed to compare different miRNA target prediction methods easily using R (http://www.r-project.org) for statistics.

## Implementation

**Fig. 1:**
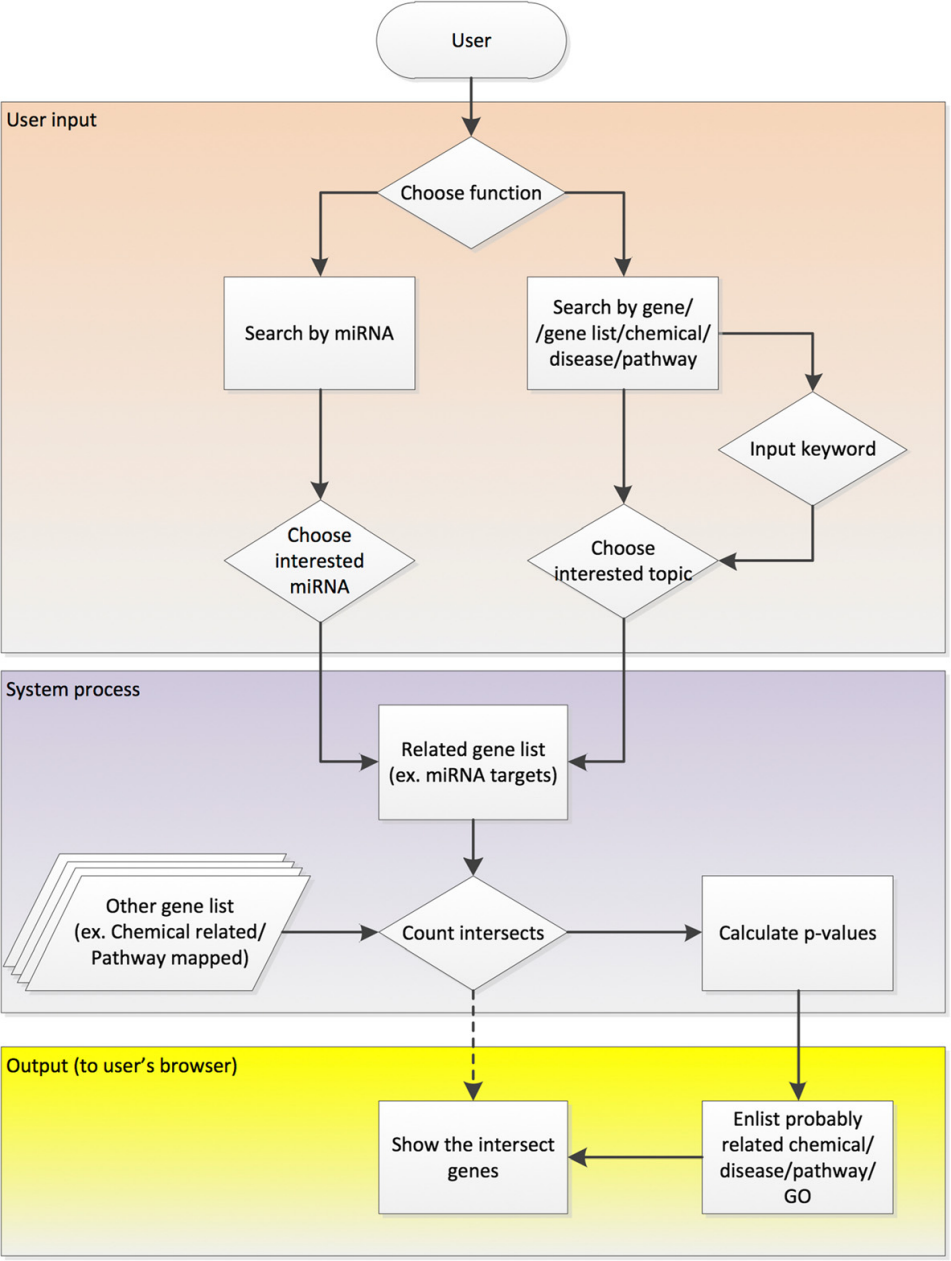
The workflow of ChemiRs web server. Illustration of six analysis modules provided by ChemiRs

The workflow of ChemiRs server is illustrated in Fig. 1. Given different types of query inputs from the users, ChemiRs server extracts relevant search results from various prediction methods and databases. Then, the results are shown in an interactive viewer and available as downloadable files. Next, the data sources, implementation and components of ChemiRs are described as follows.

### Input

To access ChemiRs web server, a user has to choose a search function from main menu for one or more searches as query processing. In the ‘Search by miRNA’ module, the user directly selects a miRNA of interest from a dropdown list of human miRNAs. For the other search modules (i.e., search by gene, genelist, chemical, disease and pathway), the user can submit a query keyword of interest to search for related topics. A graphical control checkbox permits the user to make multiple choices of both the search databases and topics of interest. Detailed descriptions of the inputs are given by scrollable tabboxes, checkboxes, radio buttons or type text. Then, the ChemiRs server processes the user query, generates the intersection of search results, and calculates the statistical significance level with *p*-value.

### Output

The search results of target genes and related associations with chemicals, diseases, pathways and GO terms are shown in the ChemiRs server. The output results are presented to the user via both an interactive viewer and downloadable files.

#### Interactive viewer

Query results are shown in a tabbox and automatically made scrollable when the sum of their width exceeds the container width size. The listbox component can automatically generate checkboxes or radio buttons for selecting list items by user selected attributes. Checkboxes allow multiple selections to be made, unlike the radio buttons. It is easy to obtain results immediately with sorting functionalities built in the grid and listbox components.

#### Downloadable files

The results can also be downloaded as comma-separated value (CSV) files, which can be easily imported into Microsoft Excel. The CSV files include all features calculated by ChemiRs. In addition, a related reference represented by the Pubmed ID is also provided. Multiple query results can also be easily integrated and exported as report documents in PDF format.

### Data sources

**Fig. 2:**
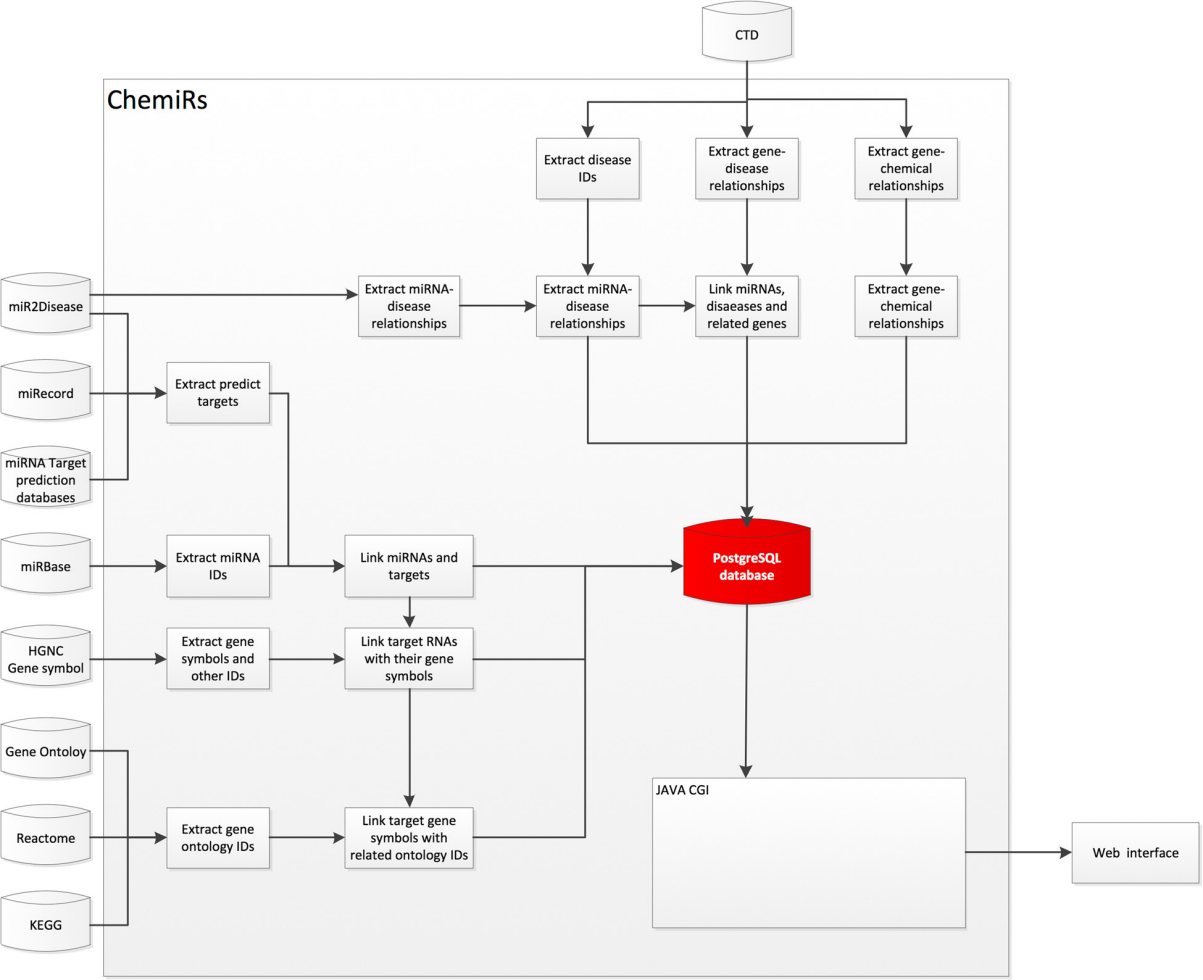
System overview of ChemiRs core framework. All results generated by ChemiRs are deposited in PostgreSQL relational databases and displayed in the visual browser and web page

Schema of the client-server architecture of ChemiRs is shown in Fig. 2. ChemiRs incorporated miRNA target prediction methods and curated databases, including DIANA-microT, miRanda, miRDB, RNAhybrid, PicTar, PITA, RNA22, TargetScan, miRWalk, miRecords, miR2Disease and miRBase as shown in Table 1. Data from the latest versions of all dependent databases are collected and integrated into a relational database in the ChemiRs server. A set of experimentally validated target genes integrated from the miRecords and mirTarBase servers is also integrated in the ChemiRs server. In addition, biological information from CTD, KEGG, REACTOME and Gene Ontology databases were manually curated into ChemiRs. The information is stored in a remote PostgreSQL server which is accessed through a Java Model-View-Controller (MVC) web service design. MyBatis library is used to connect to databases, and data can be retrieved by clients in both text and PDF formats.

**Table 1:** The versions and links of dependent databases used in the ChemiRs server

Database	Version	Link
CTD	2016/2/9	http://ctdbase.org/
miR2Disease	2011/3/14	http://www.mir2disease.org/
miRecords	2013/4/27	http://c1.accurascience.com/miRecords/
miRBase	Release 21	http://www.mirbase.org/ftp.shtml
miRWalk	2011/3/29	http://zmf.umm.uni-heidelberg.de/apps/zmf/mirwalk/
DIANA-microT	Version 4.0	http://diana.imis.athena-innovation.gr/DianaTools/index.php?r=microtv4/index
miRanda	August 2010 Release	http://www.microrna.org/microrna/home.do
miRDB	Version 5.0	http://mirdb.org/miRDB/
PicTar(4way)	2007/3/1	http://pictar.mdc-berlin.de/cgi-bin/PicTar_vertebrate.cgi
PicTar(5way)	2007/4/1	http://pictar.mdc-berlin.de/cgi-bin/new_PicTar_vertebrate.cgi
TargetScan	Version 6.0	http://www.targetscan.org/
HGNC	2016/2/29	http://www.genenames.org/cgi-bin/statistics
miRTarBase	Release 6.0	http://mirtarbase.mbc.nctu.edu.tw/index.php

## Results and discussion

### Data statistics in ChemiRs

The data statistics of ChemiRs are described in Table 2. All data were organized in ChemiRs.

**Table 2:** Data statistics in the ChemiRs server

Category	Total number
Unique miRNAs	2,588
Unique genes	36,817
Unique chemicals	161,394
Unique diseases	11,860
Unique pathways	292
Gene Ontology (GO) terms	41,468
miRNA-target genes associations	5,087,441
miRNA-disease associations	2,323
Chemical-gene interactions	500,105
Gene-disease associations	182,490
Chemical-disease associations	1,834,693
Gene-GO annotations	314,375

### Case studies

The aim of ChemiRs web server is to provide integrated and comprehensive miRNA target prediction analysis via flexible search functions, including search by miRNAs, gene lists, chemicals, genes, diseases and pathways. Next, case study examples by six different search methods are described in the following sections.

#### Search by a miRNA

**Fig. 3:**
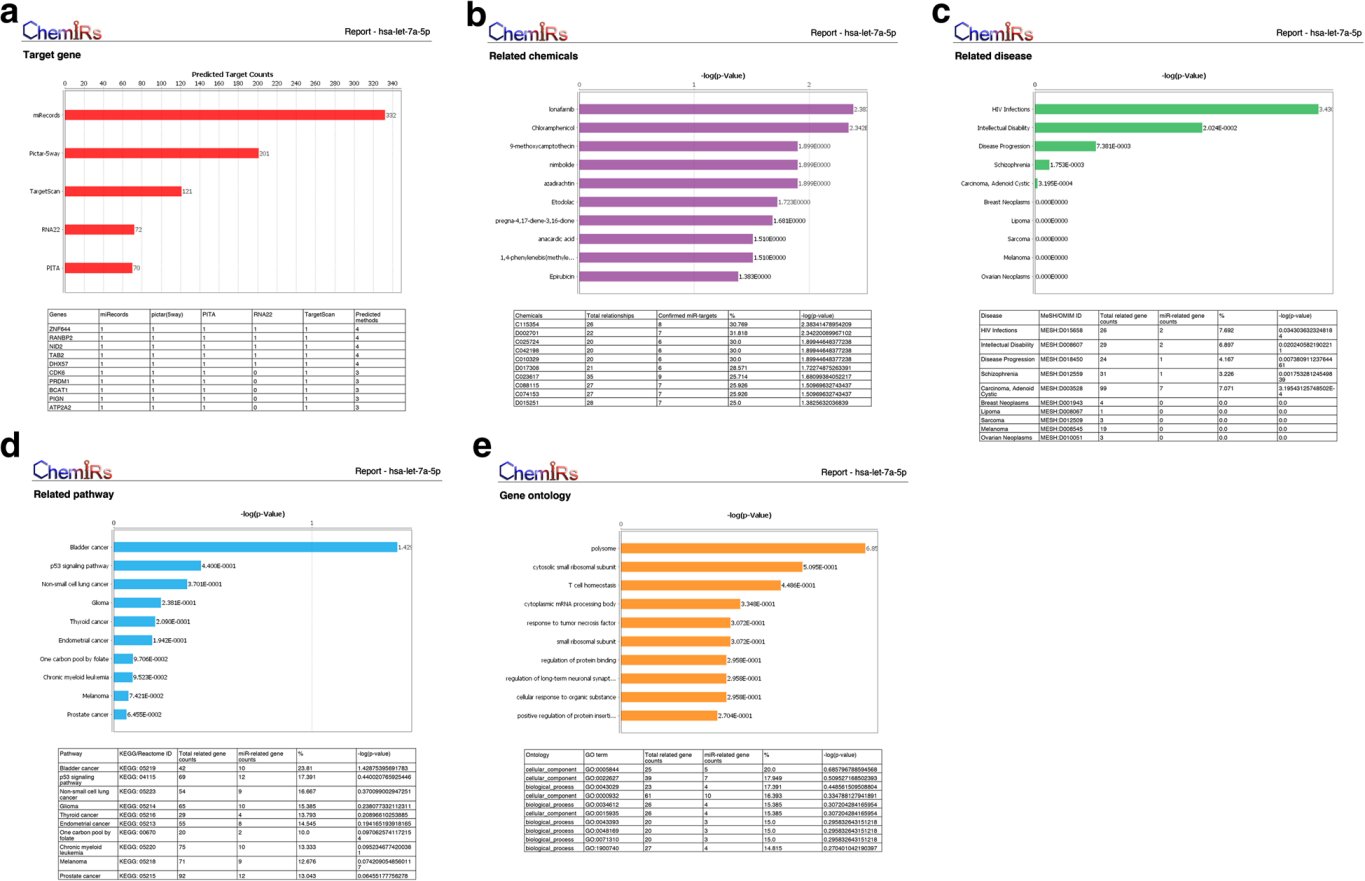
Query result of ‘hsa-let-7a-5p’ by ‘Search by miRNA’ module in ChemiRs. Given a miRNA as query, ChemiRs identifies related **a** Targets, **b** Chemicals, **c** Diseases, **d** Pathways and **e** GO terms as output, respectively

**Fig. 4:**
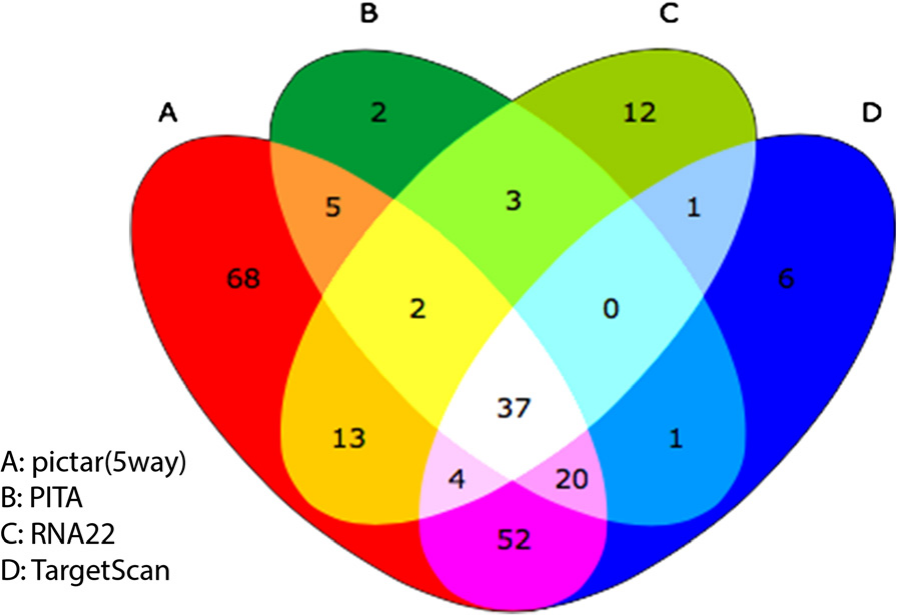
The four-way Venn diagram of hsa-let-7a-5p target genes using **a** pictar(5way), **b** PITA, **c** RNA22 and **d** TargetScan as the miRNA target prediction methods in ChemiRs

As an example, we applied ChemiRs to analyze the hsa-let-7a-5p miRNA. We selected the miRNA ‘hsa-let-7a-5p’ in ‘Search by miRNA’ module and chose ‘pictar(5way),’ ‘PITA,’ ‘RNA22,’ and ‘TargetScan’ as miRNA target prediction methods; ‘4 minimum predicted methods’ as restrictions; and ‘Targets,’ ‘Chemicals,’ ‘Diseases,’ ‘Pathways,’ and ‘GO terms’ as the output functions, respectively. This example can be referred by clicking ‘Tip#2 logical analysis’ on the start page of ChemiRs. As shown in Fig. 3, a PDF report including top ten results can be easily downloaded. We checked ‘target genes,’ the top ten ‘related chemicals,’ ‘related diseases,’ ‘related pathways,’ and ‘related GO terms’ returned by ChemiRs, which were sorted according to their significance of activity changes denoted by -log(*p*-value). The *p*-value represents the probability of a random intersection of two different gene sets, and the *p*-value calculations are based on hypergeometric distribution. The probability to randomly obtain an intersection of certain size between user’s set and a network/pathway follows hypergeometric distribution. The lower the *p*-value, the higher is the non-randomness of finding such intersection. By taking log of *p*-value, the higher the -log(*p*-value), the higher is the non-randomness. Generally, when *p*-value is considered as 0.05, the -log(*p*-value) greater than 2.995 denotes statistically significant. As shown in Fig. 4, our system identified 37 miRNAs within the intersection of the 4-way Venn diagram. Notably, the top one related pathway, ‘Bladder cancer,’ has already been reported to be associated with the hsa-let-7a miRNA in biomedical literature [28]. This demonstrates that our proposed method is able to identify important features that correspond well with biological insights.

#### Search by a gene list

We applied ChemiRs to analyze a gene list data reported by Naciff et al. [29], in which the gene set was selected according to expression changes induced by Bisphenol A (BPA) and 17alpha-ethynyl estradiol in human Ishikawa cells. We downloaded the gene list with 76 genes in Table 6 [29] under the accession number GSE17624. We used the 76 genes gene symbols as input in ChemiRs by choosing ‘Search by gene list’ module, and ‘miRNAs,’ ‘Chemicals,’ ‘Diseases,’ ‘Pathways,’ and ‘GO terms’ as the output functions; all ten methods as miRNA target prediction methods; and ‘5 minimum predicted methods’ as restrictions, respectively.

We analyzed the top ten related chemicals returned by ChemiRs, which were sorted according to their significance of activity changes (i.e., −log(*p*-value)). Interestingly, we found that these chemicals have already been well-known to be associated with estrogens or Endocrine Disrupting Chemicals (EDCs). In fact, many industrially made estrogenic compounds and other EDCs are potential risk factors of cancer. Moreover, estrogen and progesterone receptor status have already been reported to be associated with breast cancer [30]. For example, BPA was linked to breast cancer tumor growth [31]. It is expected that other chemicals might also be involved in ‘Pathways in cancer’ returned by ChemiRs, and these chemicals might be potential candidates for further investigation.

#### Search by a chemical

**Fig. 5:**
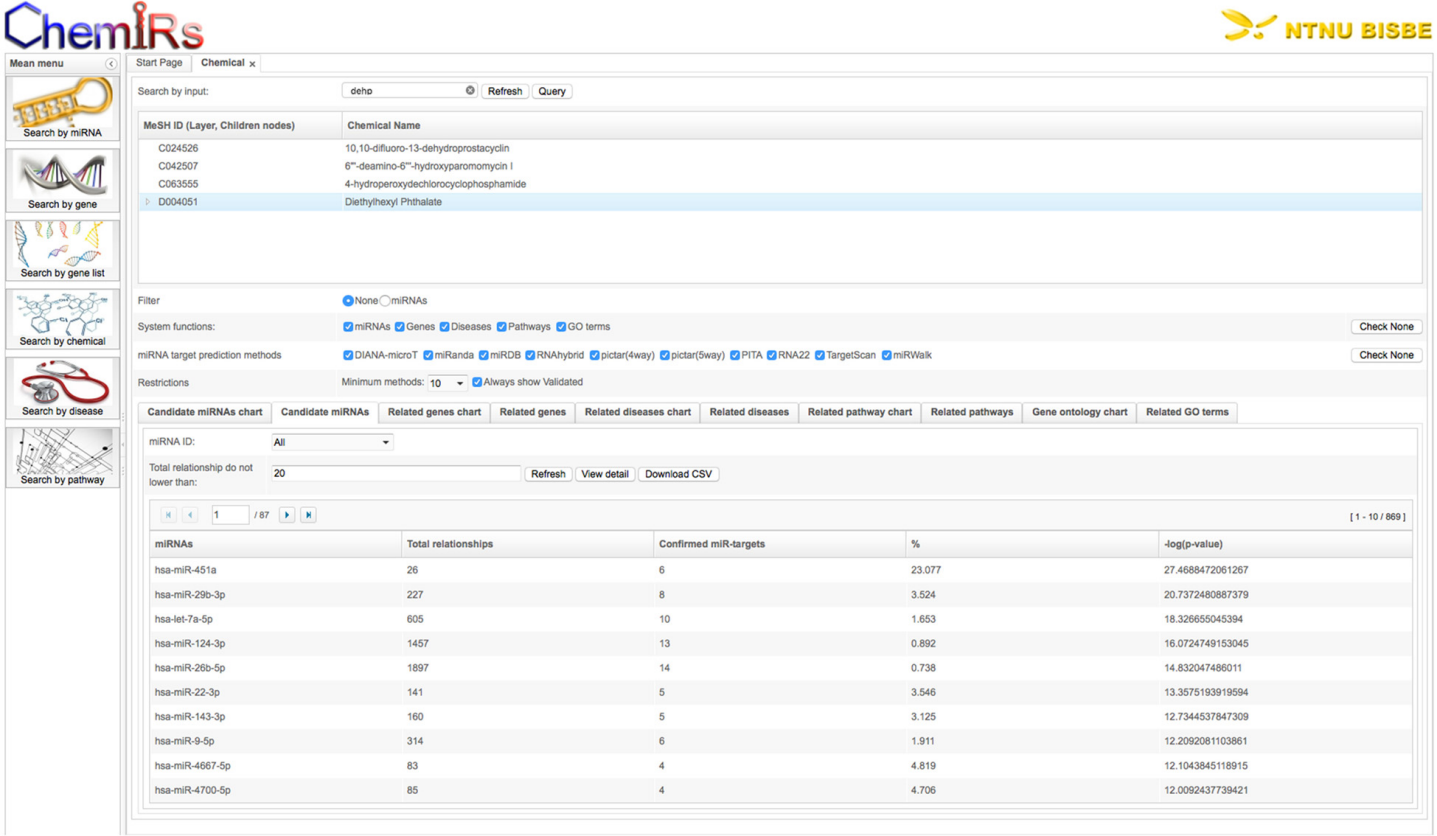
Query result of ‘DEHP’ by ‘Search by chemical’ module in ChemiRs. Related miRNAs of MeSH ID ‘D004051, Diethylhexyl Phthalate’ are listed

Here, we exemplify the application of ChemiRs to search by chemicals. We applied ChemiRs to analyze diethylhexyl phthalate (DEHP) by submitting ‘DEHP’ in ‘Search by chemical’ module. After pressing the ‘Refresh’ button, we clicked the Medical Subject Heading (MeSH) ID ‘D004051, Diethylhexyl Phthalate’ and chose ‘None’ as the filter; ‘miRNAs,’ ‘Genes,’ ‘Diseases,’ ‘Pathways,’ and ‘GO terms’ as the output functions; all ten methods as miRNA target prediction methods, and ‘10 minimum predicted methods’ as restrictions, respectively. As shown in Fig. 5, the results can be easily downloaded as CSV files.

We checked ‘Candidate miRNAs,’ the top ten ‘related genes,’ ‘related diseases,’ ‘related pathways,’ and ‘related GO terms’ returned by ChemiRs, which were sorted according to their significance of activity changes (i.e., −log(*p*-value)). The 93 related human genes and their associated references are listed in Table 3. The top one related pathway is ‘Pathways in cancer,’ and the top one related disease is ‘Brest-Ovarian Cancer, Familiar, Susceptibility To, 1; BROVCA1 (OMIM: 604370).’ DEHP is converted by intestinal lipases to mono-(2-ethylhexyl) phthalate (MEHP), which is then preferentially absorbed [2]. It has already been reported that exposure to the parent compound of the phthalate metabolite MEHP might be associated with breast cancer [32].

**Table 3:** Ninety-three related human genes and associated PubMed references of searching by chemical for MeSH ID (D004051, Diethylhexyl Phthalate)

Gene	Chemical	Reference PubMed ID
NR1I2	Diethylhexyl Phthalate	23899473;16054614;11581012;22206814;17003290;21227907
PPARG	mono-(2-ethylhexyl)phthalate	21561829;10581215;16326050;12927354;23118965
PPARA	Diethylhexyl Phthalate	10581215;20123618;21354252;16455614
CYP3A4	mono-(2-ethylhexyl)phthalate	23545481;18332045;22186153
CYP19A1	mono-(2-ethylhexyl)phthalate	22401849;19501113;19822197
ESR1	Diethylhexyl Phthalate	20382090;16756374;15840436
CYP3A4	Diethylhexyl Phthalate	11581012;18332045;21742782
CASP3	Diethylhexyl Phthalate	22155658;23220035;21864672
CASP3	mono-(2-ethylhexyl)phthalate	12927354;19165384;23360888
PPARA	mono-(2-ethylhexyl)phthalate	10581215;20123618;16326050
CYP1A1	Diethylhexyl Phthalate	8242868;16954067
NR1I3	Diethylhexyl Phthalate	21227907;23899473
NR4A1	mono-(2-ethylhexyl)phthalate	23118965;19822197
CYP2C9	mono-(2-ethylhexyl)phthalate	22186153;23545481
AR	Diethylhexyl Phthalate	19643168;20943248
AKR1B1	Diethylhexyl Phthalate	20943248;19643168
AKT1	Diethylhexyl Phthalate	19956873;23793038
IL4	Diethylhexyl Phthalate	20082445
HEXB	Diethylhexyl Phthalate	20082445
HEXA	Diethylhexyl Phthalate	20082445
ESR2	Diethylhexyl Phthalate	15840436
CYP1B1	Diethylhexyl Phthalate	16040568
CXCL8	Diethylhexyl Phthalate	23724284
CDO1	Diethylhexyl Phthalate	16223563
CASP9	Diethylhexyl Phthalate	22155658
CASP8	Diethylhexyl Phthalate	22155658
CASP7	Diethylhexyl Phthalate	21864672
BCL2	Diethylhexyl Phthalate	22155658
BAX	Diethylhexyl Phthalate	22155658
AHR	Diethylhexyl Phthalate	23220035
ACADVL	Diethylhexyl Phthalate	21354252
ACADM	Diethylhexyl Phthalate	21354252
ABCB1	Diethylhexyl Phthalate	17003290
ZNF461	mono-(2-ethylhexyl)phthalate	19822197
VCL	mono-(2-ethylhexyl)phthalate	22321834
TXNRD1	mono-(2-ethylhexyl)phthalate	23360888
TP53	mono-(2-ethylhexyl)phthalate	21515331
STAR	mono-(2-ethylhexyl)phthalate	22401849
SREBF2	mono-(2-ethylhexyl)phthalate	23118965
SREBF1	mono-(2-ethylhexyl)phthalate	23118965
SQLE	mono-(2-ethylhexyl)phthalate	23118965
SLC22A5	mono-(2-ethylhexyl)phthalate	23118965
SCD	mono-(2-ethylhexyl)phthalate	23118965
SCARA3	mono-(2-ethylhexyl)phthalate	23360888
PTGS2	mono-(2-ethylhexyl)phthalate	23360888
PRNP	mono-(2-ethylhexyl)phthalate	23360888
PPARGC1A	mono-(2-ethylhexyl)phthalate	20123618
NR4A3	mono-(2-ethylhexyl)phthalate	19822197
NR4A2	mono-(2-ethylhexyl)phthalate	19822197
NR1I2	mono-(2-ethylhexyl)phthalate	16054614
NR1H3	mono-(2-ethylhexyl)phthalate	23118965
NCOR1	mono-(2-ethylhexyl)phthalate	20123618
MYC	mono-(2-ethylhexyl)phthalate	22321834
MMP2	mono-(2-ethylhexyl)phthalate	22321834
MED1	mono-(2-ethylhexyl)phthalate	20123618
MBD4	mono-(2-ethylhexyl)phthalate	20123618
MARS	mono-(2-ethylhexyl)phthalate	22321834
LHCGR	mono-(2-ethylhexyl)phthalate	22401849
LFNG	mono-(2-ethylhexyl)phthalate	22321834
IL17RD	mono-(2-ethylhexyl)phthalate	22321834
ID1	mono-(2-ethylhexyl)phthalate	22321834
HSD11B2	mono-(2-ethylhexyl)phthalate	19786001
HMGCR	mono-(2-ethylhexyl)phthalate	23118965
GUCY2C	mono-(2-ethylhexyl)phthalate	22401849
GLRX2	mono-(2-ethylhexyl)phthalate	23360888
GJA1	mono-(2-ethylhexyl)phthalate	22321834
FSHR	mono-(2-ethylhexyl)phthalate	22401849
FSHB	mono-(2-ethylhexyl)phthalate	19501113
FASN	mono-(2-ethylhexyl)phthalate	23118965
EP300	mono-(2-ethylhexyl)phthalate	20123618
DHCR24	mono-(2-ethylhexyl)phthalate	23360888
DDIT3	mono-(2-ethylhexyl)phthalate	22321834
CYP2C19	mono-(2-ethylhexyl)phthalate	22186153
CYP1A1	mono-(2-ethylhexyl)phthalate	15521013
CTNNB1	mono-(2-ethylhexyl)phthalate	22321834
CSNK1A1	mono-(2-ethylhexyl)phthalate	16484285
CLDN6	mono-(2-ethylhexyl)phthalate	22321834
CGB	mono-(2-ethylhexyl)phthalate	22461451
CGA	mono-(2-ethylhexyl)phthalate	19501113
CELSR2	mono-(2-ethylhexyl)phthalate	16484285
CDKN1A	mono-(2-ethylhexyl)phthalate	21515331
CASP7	mono-(2-ethylhexyl)phthalate	23360888
BCL2	mono-(2-ethylhexyl)phthalate	12927354
BAX	mono-(2-ethylhexyl)phthalate	12927354
AOX1	mono-(2-ethylhexyl)phthalate	23360888
VEGFA	Diethylhexyl Phthalate	18252963
AMH	mono-(2-ethylhexyl)phthalate	19165384
TNF	Diethylhexyl Phthalate	20082445
TIMP2	Diethylhexyl Phthalate	19956873
SUOX	Diethylhexyl Phthalate	16223563
RPS6KB1	Diethylhexyl Phthalate	23793038
PPARD	Diethylhexyl Phthalate	16455614
PIK3CA	Diethylhexyl Phthalate	23793038
PAPSS2	Diethylhexyl Phthalate	16223563
PAPSS1	Diethylhexyl Phthalate	16223563
NCOA1	Diethylhexyl Phthalate	11581012
MYC	Diethylhexyl Phthalate	16455614
MTOR	Diethylhexyl Phthalate	23793038
MMP9	Diethylhexyl Phthalate	19956873
MMP2	Diethylhexyl Phthalate	19956873
MAPK3	Diethylhexyl Phthalate	16455614
MAPK1	Diethylhexyl Phthalate	16455614
LAMP3	Diethylhexyl Phthalate	20678512

#### Search by a gene

We applied ChemiRs to analyze the CXCR4 gene using ‘Search by gene’ module. After pressing the ‘Refresh’ button, we clicked ‘CXCR4,’ chose all output system functions, and pressed the ‘Query’ button. All the ‘related miRNAs,’ ‘related chemicals,’ ‘related diseases,’ ‘related pathways,’ and ‘related GO terms’ will be returned by ChemiRs.

#### Search by a disease

We applied ChemiRs to analyze Schizophrenia in ‘Search by disease’ module. We used ‘Schizophrenia’ as query and pressed the ‘Refresh’ button. A simple tree data model is used to represent a disease tree, and we pressed the light blue line’MeSH: D012559 Schizophrenia.’ All disease annotations included ‘MeSH Heading’ (i.e., controlled term in the MeSH thesaurus), ‘Tree Number’ (i.e., tree number of the MeSH term), ‘Scope Note’ (i.e., the scope notes that define the subject heading), and ‘MeSH Tree Structures’ (i.e., tree structure of the MeSH term) will be returned by ChemiRs.

#### Search by a pathway

We applied ChemiRs to analyze a cell cycle pathway using ‘Search by pathway’ module. We entered ‘cell cycle’ and pressed the ‘Refresh’ button, then five relevant pathways are listed. After we pressed the light blue line ‘KEGG: 04110 Cell cycle,’ all the hsa04110 pathway information will be returned.

### Future extensions

In the future, we will continuously develop and enhance the interactive analysis module and adjust the web service for better user-experience. An automatic update will also be carried out monthly to keep pace with the latest database versions. It is also planned to incorporate more applications for gene expression data and allow users to customize their own visualization.

## Conclusion

The ChemiRs web server integrates and compares ten miRNA target prediction methods of interest. The server provides comprehensive features to facilitate both experimental and computational target predictions. In addition, ChemiRs incorporates flexible search modules including (i) search by miRNA, (ii) search by gene, (iii) search by gene list, (iv) search by chemical, (v) search by disease and (vi) search by pathway. Moreover, ChemiRs can make predictions for Homo sapiens miRNAs of interest, and also allow fast search of query results for multiple miRNA selection and logical restriction, which can be easily integrated and exported as report documents in PDF format. The service is unique in that it integrates a large number of miRNA target prediction methods, experiment results, genes, chemicals, diseases and GO terms with instant and visualization functionalities.

## Availability and requirements

Home page: http://omics.biol.ntnu.edu.tw

Tip: http://omics.biol.ntnu.edu.tw: Welcome

Demo: http://omics.biol.ntnu.edu.tw: Video

Tutorial: http://omics.biol.ntnu.edu.tw: Help

Operating system(s): Both portal and clients are platform independent.

Programming language: JAVA, JavaScript

Any restrictions to use by non-academics: None

## Abbreviations

BPA: bisphenol A
DEHP: diethylhexyl phthalate
GO: gene ontology
MEHP: mono-(2-ethylhexyl) phthalate
MeSH: medical subject heading
miRNA: microRNA
MVC: Model-View-Controller
